# MOVING FORWARD TOWARD DEPLOYING HEALTH INFORMATION TECHNOLOGY IN ALL US NURSING HOMES

**DOI:** 10.1093/geroni/igae098.1218

**Published:** 2024-12-31

**Authors:** Gregory Alexander, Christina Caraballo, Maria Moen, Cynthia Morton, Terrance O’Malley

**Affiliations:** Columbia University, New York, New York, United States; HIMSS, Chicago, Illinois, United States; BA, Richardson, Texas, United States; Advion, Washington, District of Columbia, United States; Harvard Medical School/Massachusetts General Hospital, Boston, Massachusetts, United States

## Abstract

The Moving Forward Coalition Health Information Technology (HIT) Committee has refined two action plans designed to promote HIT adoption in all U.S. nursing homes (NH). In this work, Moving Forward is responding to two recommendations in the (National Academies of Sciences, Engineering, Medicine Report of 2022 (The National Imperative to Improve Nursing Home Quality: Honoring Our Commitment to Residents, Families, and Staff) to deploy HIT in all NHs and to ensure that care is consistent with the individual’s goals, preferences, and priorities (GPPS). The first plan includes a HIT Readiness Guide to inform the adoption of the essential HIT capabilities required for survival in Value Based Payment models (VBP) mandated by the Centers for Medicare & Medicaid Services (CMS) to begin in 2030. The second provides an HIT enabled tool to facilitate the collection of an individual’s GPPs to facilitate goal concordant care consistent with an individual’s GPPs and to enable measurement of the degree to which the care provided is concordant with those GPPS. In this presentation, members of the HIT Committee will discuss the “what, why, and how” of these plans and the progress to date. Additionally, panel members will discuss plans for testing and evaluating tools developed from our initial work. Included will be a discussion of the challenges of sustainability and feasibility in the face of little federal support for these efforts. Although the Moving Forward Coalition and the NASEM report focus on NHs, the HIT tools being developed here will benefit all sites of care.

